# The usefulness of DNA derived from third stage larvae in the detection of *Ashworthius sidemi* infection in European bison, by a simple polymerase chain reaction

**DOI:** 10.1186/1756-3305-7-215

**Published:** 2014-05-08

**Authors:** Bożena Moskwa, Justyna Bień, Katarzyna Goździk, Władysław Cabaj

**Affiliations:** 1 Witold Stefański Institute of Parasitology of the Polish Academy of Sciences, ul. Twarda 51/55, 00-818 Warsaw, Poland

**Keywords:** *Ashworthius sidemi*, L3 DNA, PCR, *Bison bonasus*

## Abstract

**Background:**

*Ashworthius sidemi*, a blood-sucking nematode, is a primary parasite of Asiatic cervides, primarily sika deer (*Cervus nippon*). As *A. sidemi* infections are common in bison, red and roe deer, and gastrointestinal nematodes are often exchanged between animals, it is possible that other farm animals such as cows and sheep that may use the same pastures can be infected. Hence, histopathological changes observed in the walls of the abomasa and duodena of infected wildlife caused by a strong parasite presence may become an important health problem also for farm animals.

**Methods:**

In the present study, a simple PCR test for the detection of *A. sidemi* infection in European bison based on DNA from third stage infective larvae (L3) has been optimized.

**Results:**

The species-specific primers generated a 406 bp fragment, and *A. sidemi* DNA could be detected at concentrations of 0.1 pg/μl. The specificity of PCR was confirmed by the use of the genomic DNA of adult *Ostertagia ostertagi, Haemonchus contortus, Cooperiaoncophora* as negative controls.

**Conclusion:**

It is possible to detect *A. sidemi* infection in European bison using DNA from L3. If this nematode infection is transmitted to cows this method may be effective to diagnose invasion in breeding animals *in vivo*.

## Background

Parasitic helminth infections cause production losses in farm animals worldwide. In addition, it has been well established that parasitic gastrointestinal (GI) helminth infection is commonplace and has similar impacts on performance and fitness in wild animals [1,2].

The European bison (*Bison bonasus bonasus* L.), the largest herbivorous animal in Europe is protected both by international and national laws. Most European bison live in Poland: the largest single population, of 500 head, inhabits the Białowieża Forest, the last ancient primeval woodland in Europe. This herd plays an important role in the restitution and protection program of the species, and in the last decade, many of the bison have been transported from Białowieża to other locations in Poland, as well as other European countries including Spain, Denmark, Germany and Sweden, to create new herds, both living in enclosures and in the wild [3-6]. As European bison are grazing animals, and their health is often at risk by being constantly exposed to GI nematode infection, the helminth fauna of this animal species in Poland has been studied in detail over the last 40 years [7-10].

*Ashworthius sidemi*, a blood-sucking nematode of the Trichostrongylidae family, is a primary parasite of Asiatic cervides, particularly sika deer (*Cervus nippon*). The introduction of this host species in Ukraine, Slovakia, the Czech Republic and France [11-13] has also allowed the parasite to spread throughout the area and its neighboring countries. Sika deer were first introduced to Poland in 1895 near Pszczyna, Silesia, and then again in 1910, near the Vistula lagoon in the vicinity of Kadyny. The results note [14] the presence of 12 species in the helminth fauna of sika deer from the forestry division near Pszczyna, with the most common ones being *Spiculopteragia spiculoptera, S. asymetrica, Rinadia mathevossiani, Cooperia punctate, Oesophagostomum venulosum, Haemonchus contortus, Fasciola hepatica* and *Setaria* spp*.* However, neither *A. sidemi* nor any other typical parasites of sika deer were found. The authors conclude that the sika deer introduced in Poland have lost their original parasites while acquiring new species of parasitic nematodes from the native deer.

In the last decade, *A. sidemi* has been found in the helminth fauna of wild ruminants living in Polish territory: some regions being the Bieszczady Mountains and the Polish part of the Białowieża Forest, as well as the Knyszyńska, Dulowska and Augustowska Forests [8,15-18].

Ashworthiosis infection has gradually become more widespread in European bison over the years [17]. Histopathological examinations show infiltrations of inflammatory cells, mainly lymphoid cells and eosinophils, in the walls of the abomasa and duodena at various levels of intensity, as well as hyperanemiae, oedemae and mucosal lesions and proliferations of lymphatic follicles. The intensity of histopathological changes was connected to a considerable degree with the developmental stage of *A. sidemi*[10]. Mechanical injuries were often accompanied by epithelial cell dysplasia, atrophy or hyperplasia of glands and the presence of parasites in the lumen or walls of the abomasum/duodenum. Lymphatic follicle proliferation was observed in regional lymph nodes, as well as the presence of eosinophils and destruction of reproduction centers.

The diagnosis of GI nematodes still relies predominantly on coproscopy using microscopic examination. Until now, both the presence and the number of *A. sidemi* specimens in the abomasum and duodena in wildlife was confirmed during post mortem examination only. To facilitate an easier and more reliable diagnosis, a simple polymerase chain reaction (PCR)-based technique was developed to differentiate *A. sidemi*. This method may be also useful to diagnose *A. sidemi* invasion in breeding animals *in vivo*. Hence, the aim of the study was to optimize a simple PCR test for the detection of *A. sidemi* infection in European bison using DNA from third stage infective larvae (L3).

## Methods

### Parasitological measurement

The study was performed on European bison immobilized in the summer of 2012 in the Bieszczady Mountains, Poland.

The faecal samples were collected directly from the rectum. Faecal pallets (30 g) were placed in Petri dish cultures and incubated at 25°C for 7-10 days. The cultures were examined using a stereomicroscope. A mixed population of infective larvae visible in the peripheral part of the culture were transferred to Eppendorf tubes. The samples were stored at -20°C until molecular species identification.

**Figure 1:**
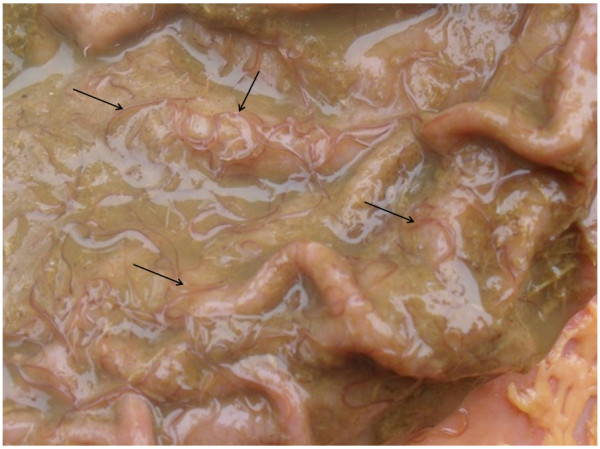
**The adult *A. sidemi *** **visible in the abomasum of European bison.**

In addition, the abomasa and duodena of European bison shot during the selective hunting seasons in the Białowieża Forest in winter 2012 were examined for the presence of worms (Figure 1). Bison shooting was carried out according to the direction of the Director General of Environment Protection (DOP-OZGIZ).

**Figure 2:**
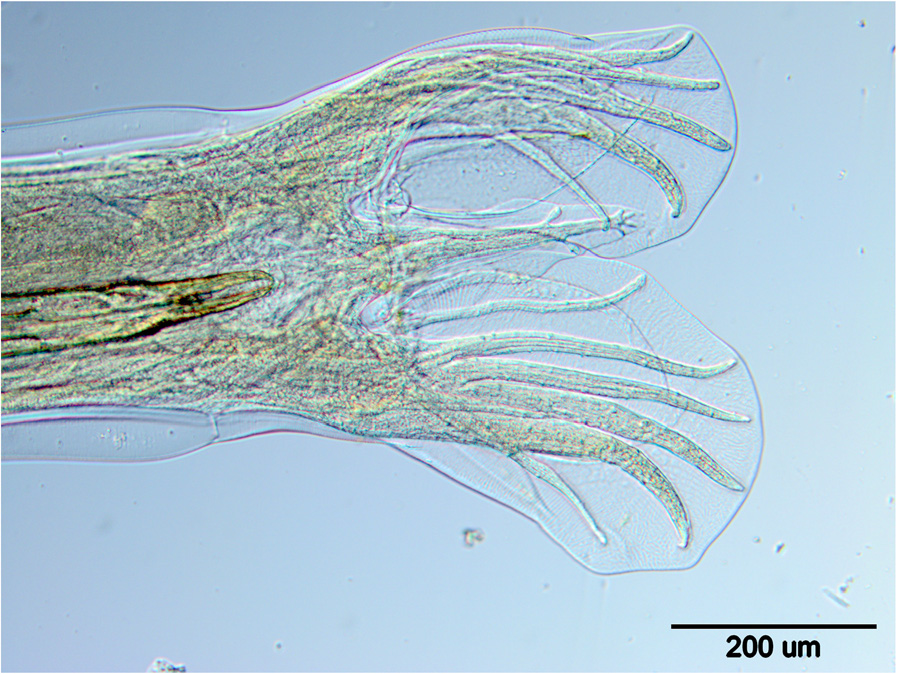
***A. sidemi*: posterior end of male.**

**Figure 3:**
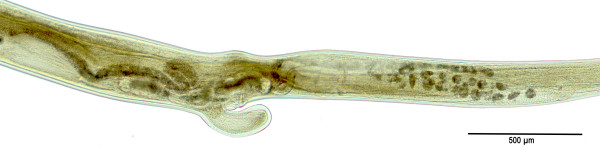
***A. sidemi*: vulvar region of female.**

The shot animals were necropsied within a few hours after death using the repeated decantation method. The sediment was then placed partially onto the Petri dishes and examined by stereomicroscope. Using an optical microscope nematodes were identified at the species level based on morphological and morphometric parameters published previously [8] (Figures 2 and 3). The nematodes were carefully washed in PBS and stored at -20°C for further analysis.

### DNA isolation

Genomic DNA was extracted from L3 mixtures and from morphologically identified adult parasites using a Nucleospin Tissue DNA extraction kit (Macherey-Nagel, Germany), according to the manufacturer’s instructions. DNA from adult *A. sidemi* was used as a control.

The samples were examined for *A. sidemi* DNA using a species-specific PCR method [19,20]. Firstly, a fragment spanning the 3′ -end of the ssrRNA to the 5′ –end of the lsrRNA gene was amplified and sequenced (GenBank accession number EF467325). Next, species-specific primers were designed based on the deposited sequence (*A. sidemi* R specific: 5′-ACA ACA TTA ACA CCT GTT GCA TGT-3′; *A. sidemi* F specific: 5′-ACT GTA TCC GAA TAT ATA TCG GAG-3′). These were directed to unique sequences in the internal transcriber spacer (ITS1 and ITS2) to generate a 406 bp fragment.

The reagents used for the PCR reaction were purchased from Fermentas (MBI Fermentas, USA). The PCR reaction itself was performed in a final volume of 25 μl containing 10 pM of each primer, 1 U Taq DNA polymerase (Fermentas), and 1 μl DNA. The amplifications were carried out with an initial denaturation step of 95°C for 5 min, followed by 35 cycles of denaturation at 94°C for 45 s, annealing at 52°C for 45 s, extension at 72°C for 1 min, and a final extension of 72°C for 10 min., all carried out in a TC-512thermocycler (Techne, USA).

The amplified genomic DNA samples were analyzed on 2% agarose gels stained with GelRed (Biotium) in TAE buffer, at 100 V. The gels were visualised and analysed under UV light using the KODAK 1D™ Electrophoresis Documentation and Analysis System.

To determine the sensitivity of the PCR amplicons of the examined samples, the DNA concentration was determined using a NanoDrop ND 1000 spectrophotometer. A dilution series from 0.1 ng/ml down to 0.1 pg/ml was used in the study. The genomic DNA of adult *Ostertagia ostertagi, H. contortus, Cooperiaoncophora* were used as a negative control for the PCR reaction.

A segment of approximately 406 bp was excised from agarose gel and purified using the NucleoSpin Extract II (Macherey-Nagel, Germany). DNA was sequenced in the forward and reverse directions by Genomed (Warsaw, Poland). The sequences were edited using Vector NTI Advance™, version 10 (Invitrogen, USA). BLAST searches were performed in order to compare the sequence with those in GenBank.

## Results and discussion

Grazing animals are constantly exposed to GI nematode infection, and this exerts a major effect on animal production throughout the world. The economic losses are mainly caused by a significant decrease in the condition of the animals, leading to reduced fertility and the frequent deaths of infected animals.

The diagnosis of GI nematodes still relies predominantly on coproscopy using microscopic examination. Different strongylid parasite species differ substantially in their pathogenicity and so require different interpretations of faecal egg counts. In addition, faecal egg counts cannot be directly correlated with worm burdens, as they could not be identified to genus or species [21]. However, direct PCR based on DNA delivered from purified trichostrongylid egg suspension has been previously described as being suitable for identification at the genus level [22-25], but the method has never been widely used by research groups [26]. In many cases, when the number of eggs in the faeces is low, *in vitro* culture of GI nematode eggs up to the L3 greatly. It is worthy to add that the standard procedure of L3 differentiation is time consuming and requires experienced investigators and, due to faint morphological characteristics, is not always reliable [22].

**Figure 4:**
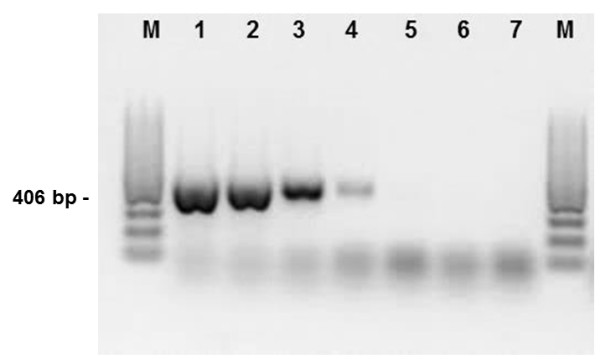
**Agarose gel (2%) electrophoresis of the products obtained in PCR experiments.** DNA isolated from adult *A. sidemi* was used as a template. A limited dilution series from 1 ng/μl (line 1) to 0.1 pg/μl (line 5). A sample containing 0.1 pg/μl (line 5) was clearly visible on the original gel. Lane M, molecular size marker (100 bp DNA ladder, Fermentas).

The present studies aimed to optimise the PCR technique for the GI nematode *A. sidemi* detection based on genomic DNA extracted from L3 mixtures. The species-specific primers generated a 406 bp fragment and *A. sidemi* DNA could be detected at a concentration of 0.1 pg/μl, thus demonstrating the sensitivity of the designed PCR system (Figure 4).

A diagnostic PCR technique for *A. sidemi* proposed earlier [19,20] has been included in a multiplex PCR assay capable of detecting four important GI species simultaneously (*H. contortus, O. ostertagi, C. oncophora, A. sidemi*). The method was optimised for DNA obtained from the adult parasite and could be used to classify species identified morphologically after necropsy.

The results of the studies reveal that *A. sidemi*, a nematode in the Trichostrongylidae family is widespread in wild ruminants such as red deer, roe deer, fallow deer, moose and European bison in Poland [15-17]. Parasitological necropsies of European bison have revealed very high infection intensities, reaching a maximum value of 77,630 specimens [17]. Histopathological examination suggests that the presence of such a large number of parasites and its associated histopathological changes have a significant impact on the health of the bison [10].

The European bison population in Białowieża Forest is an important part of the restitution and protection program of the species. However, the establishment of new herds of bison living in enclosures or in the wild, which are derived from the animals in the Białowieża Forest represents an additional route for the spread of the parasite in the environment.

Severe *A. sidemi* infection is known to have a considerable influence on the health status and conservation of the European bison. The high pathogenicity observed of *A. sidemi* infections has highlighted the need to develop a method for species identification, which can be used *in vivo*.

**Figure 5:**
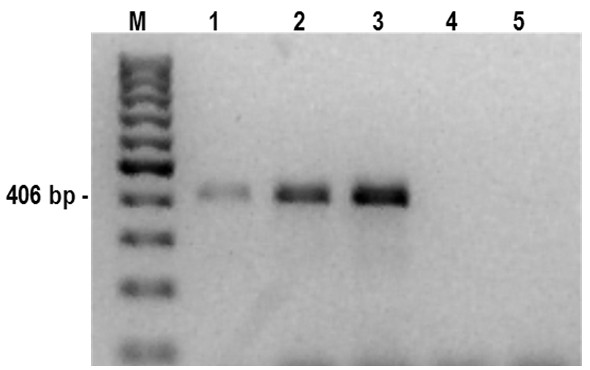
**Agarose gel (2%) electrophoresis of the products obtained in PCR experiments: line 1-2 DNA samples from L3 *A. sidemi*, 3- DNA samples from adult *A. sidemi*, 4-5 negative control, Lane M, molecular size marker (100 bp DNA ladder, Fermentas).**

**Figure 6:**
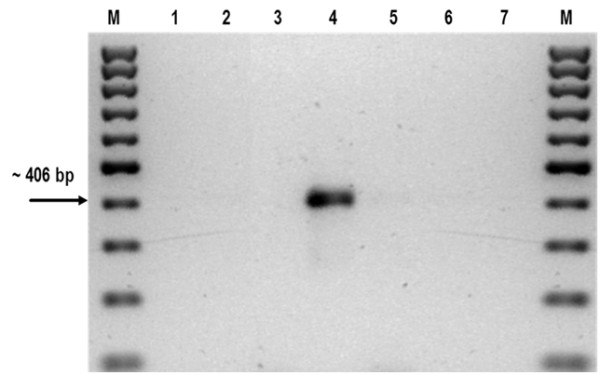
**Agarose gel (2%) electrophoresis of the products obtained in PCR experiments.** DNA samples from adult: line 1- *C. oncophora*, 2- *O. ostertagi*, 3- *H. contortus*, 4- *A. sidemi* 5- negative control (*Bison bonasus* muscle sample) 6-7 negative control (H_2_0), M, molecular size marker (100 bp DNA ladder, Fermentas).

The results of the present study reveal that a simple PCR test for *A. sidemi* identification based on DNA from L3 larvae allows infection in European bison to be confirmed or excluded *in vivo*, without the need to perform post-mortem examinations. The species-specific primers generated a 406 bp fragment characteristic of *A. sidemi* (Figure 5). The specificity of the presented PCR system was confirmed by the use of the genomic DNA of adult *O. ostertagi, H. contortus, Cooperiaoncophora* as a negative control (Figure 6). The sequences from *A. sidemi* L3 larvae obtained in this study have been deposited in GenBank under the accession number KF414629. They have been matched with sequences already published in GenBank, showing 99% similarity with the *A. sidemi* small subunit ribosomal RNA gene, partial sequence; internal transcribed spacer 1, 5.8S ribosomal RNA gene, and internal transcribed spacer 2, complete sequence; and large subunit ribosomal RNA gene, partial sequence (accession numbers EF467325).

It has been reported that the GI nematode fauna of the bison living in the Bieszczady Mountains has increased by 6 species characteristic of the Cervidae family compared with that of the bison living in enclosed reserves [9]. The authors conclude that the new parasite species were acquired from red deer and roe deer, and a danger exists of the spread of these parasites across Poland. Additionally, it has been suggested that possibly infections may be transmitted between wildlife and livestock, particularly sheep and cows, which may be grazed on the same pastures [15]. This hypothesis is supported by the reports of earlier experimental *A. sidemi* infection in sheep [27].

It is well established that parasitic helminth infections have similar impacts on the health status of wild and domestic animals [2,21] and the effects are often subclinical, such as reduced appetite and food assimilation, with consequences related to growth, reproduction and lactation. If *A. sidemi* infection is transmitted to cows [15], it will threaten their health status. Rapid parasite identification by PCR based on DNA from *A. sidemi* L3 larvae will allow the use of appropriate antiparasitic prophylaxis to improve their health.

## Conclusion

The results of the present study reveal that a simple PCR test for *A. sidemi* identification based on DNA from third stage infective larvae allows infection in European bison to be confirmed or excluded *in vivo*, without the need to perform post-mortem examinations. If *A. sidemi* infection is transmitted to cows this method may be effective to diagnose invasion in breeding animals *in vivo*.

## Competing interests

The authors declare that they have no competing interests.

## Authors’ contributions

BM conceived and designed the study area, JB, KG performed experiments, BM, JB analysed data, BM, JB and WC wrote the manuscript. All authors read and approved the final version of the manuscript.
